# Subthalamic nucleus physiology is correlated with deep brain stimulation motor and non-motor outcomes

**DOI:** 10.1093/braincomms/fcad268

**Published:** 2023-10-18

**Authors:** Mikael Levy, Mika Zurawel, Vincent d’Hardemare, Anan Moran, Fani Andelman, Yael Manor, Jacob Cohen, Moshe Meshulam, Yacov Balash, Tanya Gurevich, Itzhak Fried, Hagai Bergman

**Affiliations:** Movement Disorders Unit, Tel Aviv Sourasky Medical Center, Tel Aviv 6423906, Israel; Department of Neurosurgery, Tel Aviv Sourasky Medical Center, Tel Aviv 6423906, Israel; School of Medicine, Tel Aviv University, Tel Aviv 6997801, Israel; Sagol School of Neuroscience, Tel Aviv University, Tel Aviv 6997801, Israel; The Edmond and Lily Safra Center for Brain Sciences, The Hebrew University of Jerusalem, Jerusalem 9190401, Israel; Movement Disorders Unit, Tel Aviv Sourasky Medical Center, Tel Aviv 6423906, Israel; School of Medicine, Tel Aviv University, Tel Aviv 6997801, Israel; Sagol School of Neuroscience, Tel Aviv University, Tel Aviv 6997801, Israel; Department of Neurosurgery, Hospital Foundation Rothschild, Paris 75019, France; Sagol School of Neuroscience, Tel Aviv University, Tel Aviv 6997801, Israel; School of Neurobiology, Biochemistry & Biophysics, George S. Wise Faculty of Life Science, Tel-Aviv University, Tel Aviv 6423906, Israel; Movement Disorders Unit, Tel Aviv Sourasky Medical Center, Tel Aviv 6423906, Israel; School of Medicine, Tel Aviv University, Tel Aviv 6997801, Israel; Sagol School of Neuroscience, Tel Aviv University, Tel Aviv 6997801, Israel; Movement Disorders Unit, Tel Aviv Sourasky Medical Center, Tel Aviv 6423906, Israel; School of Medicine, Tel Aviv University, Tel Aviv 6997801, Israel; Sagol School of Neuroscience, Tel Aviv University, Tel Aviv 6997801, Israel; Department of Otolaryngology Head and Neck Surgery, Rambam Health Care Campus, Haifa 3525408, Israel; Movement Disorders Unit, Tel Aviv Sourasky Medical Center, Tel Aviv 6423906, Israel; School of Medicine, Tel Aviv University, Tel Aviv 6997801, Israel; Sagol School of Neuroscience, Tel Aviv University, Tel Aviv 6997801, Israel; Movement Disorders Unit, Tel Aviv Sourasky Medical Center, Tel Aviv 6423906, Israel; School of Medicine, Tel Aviv University, Tel Aviv 6997801, Israel; Sagol School of Neuroscience, Tel Aviv University, Tel Aviv 6997801, Israel; Movement Disorders Unit, Tel Aviv Sourasky Medical Center, Tel Aviv 6423906, Israel; School of Medicine, Tel Aviv University, Tel Aviv 6997801, Israel; Sagol School of Neuroscience, Tel Aviv University, Tel Aviv 6997801, Israel; Movement Disorders Unit, Tel Aviv Sourasky Medical Center, Tel Aviv 6423906, Israel; Department of Neurosurgery, Tel Aviv Sourasky Medical Center, Tel Aviv 6423906, Israel; School of Medicine, Tel Aviv University, Tel Aviv 6997801, Israel; Sagol School of Neuroscience, Tel Aviv University, Tel Aviv 6997801, Israel; The Edmond and Lily Safra Center for Brain Sciences, The Hebrew University of Jerusalem, Jerusalem 9190401, Israel

**Keywords:** Parkinson’s disease, deep brain stimulation, microelectrode recording, subthalamic nucleus, symptoms

## Abstract

Subthalamic nucleus deep brain stimulation is commonly indicated for symptomatic relief of idiopathic Parkinson’s disease. Despite the known improvement in motor scores, affective, cognitive, voice and speech functions might deteriorate following this procedure. Recent studies have correlated motor outcomes with intraoperative microelectrode recordings. However, there are no microelectrode recording–based tools with predictive values relating to long-term outcomes of integrative motor and non-motor symptoms. We conducted a retrospective analysis of the outcomes of patients with idiopathic Parkinson’s disease who had subthalamic nucleus deep brain stimulation at Tel Aviv Sourasky Medical Centre (Tel Aviv, Israel) during 2015–2016. Forty-eight patients (19 women, 29 men; mean age, 58 ± 8 years) who were implanted with a subthalamic nucleus deep brain stimulation device underwent pre- and postsurgical assessments of motor, neuropsychological, voice and speech symptoms. Significant improvements in all motor symptoms (except axial signs) and levodopa equivalent daily dose were noted in all patients. Mild improvements were observed in more posterior-related neuropsychological functions (verbal memory, visual memory and organization) while mild deterioration was observed in frontal functions (personality changes, executive functioning and verbal fluency). The concomitant decline in speech intelligibility was mild and only partial, probably in accordance with the neuropsychological verbal fluency results. Acoustic characteristics were the least affected and remained within normal values. Dimensionality reduction of motor, neuropsychological and voice scores rendered six principal components that reflect the main clinical aspects: the tremor-dominant versus the rigidity–bradykinesia-dominant motor symptoms, frontal versus posterior neuropsychological deficits and acoustic characteristics versus speech intelligibility abnormalities. Microelectrode recordings of subthalamic nucleus spiking activity were analysed off-line and correlated with the original scores and with the principal component results. Based on 198 microelectrode recording trajectories, we suggest an intraoperative subthalamic nucleus deep brain stimulation score, which is a simple sum of three microelectrode recording properties: normalized neuronal activity, the subthalamic nucleus width and the relative proportion of the subthalamic nucleus dorsolateral oscillatory region. A threshold subthalamic nucleus deep brain stimulation score >2.5 (preferentially composed of normalized root mean square >1.5, subthalamic nucleus width >3 mm and a dorsolateral oscillatory region/subthalamic nucleus width ratio >1/3) predicts better motor and non-motor long-term outcomes. The algorithm presented here optimizes intraoperative decision-making of deep brain stimulation contact localization based on microelectrode recording with the aim of improving long-term (>1 year) motor, neuropsychological and voice symptoms.

## Teaser

In this retrospective analysis, Levy *et al.* show that localization of deep brain stimulation microelectrodes implanted in the subthalamic nucleus of patients with Parkinson’s disease is correlated with change in motor and non-motor symptoms. The analysis suggests an algorithm to optimize intraoperative decision-making of deep brain stimulation contact localization to improve symptoms.

## Graphical Abstract

**Graphical Abstract:**
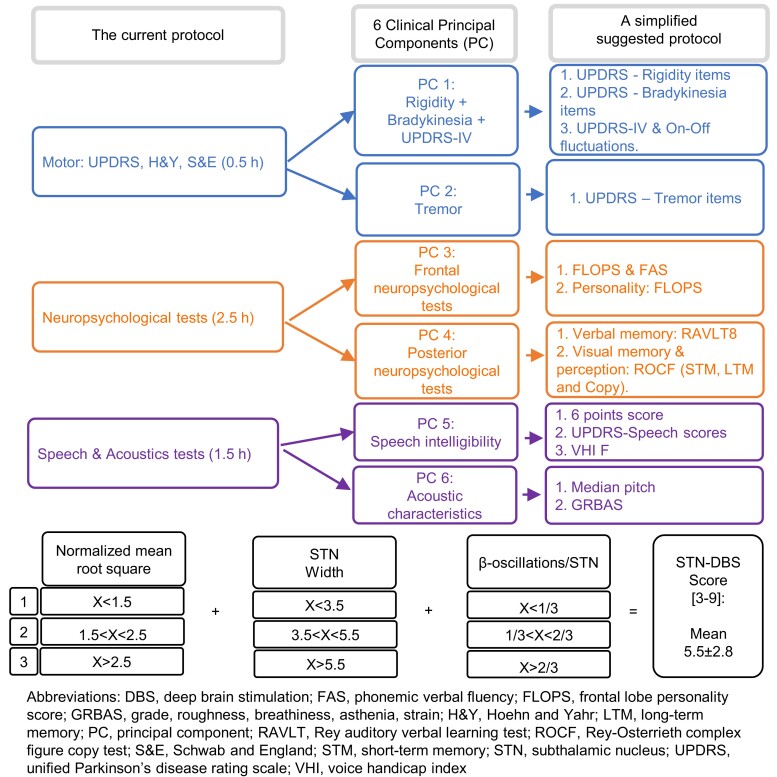

## Introduction

Since early studies on non-human primates^1,2^ and human,^3-5^ subthalamic nucleus deep brain stimulation (STN-DBS) has become a common treatment for idiopathic Parkinson’s disease. However, along with significant motor improvement, deterioration in executive functions (verbal fluency)^6,7^ and affective disorders (depression, mania, anxiety and apathy)^8,9^ has been reported after STN-DBS. In addition, STN-DBS also affects acoustic characteristics and speech intelligibility, causing hypokinetic dysarthria, hypophonia, hypoprosodia, hoarseness and impaired articulation.^10-13^

STN-DBS should precisely target the motor posterior-dorso-lateral region of the STN while avoiding its limbic (ventral) and cognitive (central) regions.^5,13-19^ Microelectrode recording (MER) improves the definition of the STN borders and its adjacent structures on sub-millimetric scales.^20,21^ Beta oscillations within the dorso-lateral oscillatory region (DLOR), which commonly occur in Parkinson’s disease, are correlated with motor outcomes^22^ but physiological correlations with neuropsychological, voice and speech outcomes are lacking.

The objective of this study was to suggest an MER-based algorithm that optimizes intraoperative decision-making concerning the proposed localization of the active lead. Such an algorithm should have a predictive value for motor, neuropsychological, voice and speech outcomes following STN-DBS.

## Materials and methods

### Study design

We conducted a retrospective analysis of the outcomes of patients with idiopathic Parkinson’s disease who had DBS-STN at Tel Aviv Sourasky Medical Centre (Tel Aviv, Israel) during 2015–2016. Patients with implanted DBS devices in locations other than the STN or those with post-STN-DBS hardware complications (two patients) were excluded. The study was approved by the institutional ethics committee (no.: 0094/11).

Prior to the procedure, each patient underwent assessments of motor, neuropsychological, voice and speech scores. STN-DBS lead placement was performed under local analgesia on awake patients and included intraoperative MER and neurological testing. DBS device implantation was done during the same surgery. Outcomes were evaluated at least 12 months following the surgery and after it was determined that the patient had achieved a steady state. MERs were analysed off-line and correlated with the patients’ motor, cognitive, emotional and speech scores and with the principal component (PC) results.

### Clinical scores

Motor symptoms were evaluated by the unified Parkinson’s disease rating scale (UPDRS) and by the Hoehn and Yahr (H&Y) scale. Both the UPDRS-I–IV sub-scales and symptom stratification into tremor, rigidity, limb bradykinesia, axial bradykinesia, dyskinesia, on–off fluctuations, speech and mentation were used. We used a conventional protocol where the steady-state parameters after both unilateral and bilateral UPDRS are defined following unilateral and bilateral stimulation, respectively. Activities of daily living (ADL) were assessed using the Schwab and England (S&E) scale.

Neuropsychological symptoms were evaluated by the Trail Making Test (TMT) Parts A & B for assessing attention and cognitive flexibility (TMT-A and TMT-B, respectively),^23^ the WAIS Digit Span Subtest for evaluating working memory and attention, the Frontal Lobe Personality Scale (FLOPS),^24^ the Wisconsin Card Sorting Test (WCST),^25^ phonemic verbal fluency (FAS)^26^ and semantic verbal fluency (using the category ANIMALS) for evaluating executive functions, the Rey–Osterrieth Complex Figure Test (ROCF)^27^ and the Hooper test for evaluating visuospatial function,^28^ the ROCF^29^ for evaluating visual memory and the Rey Auditory Verbal Learning Test (RAVLT)^29^ for evaluating verbal learning and memory. All of the neuropsychological scales are provided in detail in Supplementary Appendix 1.

The acoustic characteristics were assessed using computerized analyses of voice recordings with the PRAAT software. These included analyses of jitter (for frequency perturbations), shimmer (for amplitude perturbations), median pitch (for fundamental frequency), mean phonation time (MPT), and noise to harmonic ratio (NHR).^30^ Voice quality was evaluated by speech language pathologists using grade, roughness, breathiness, asthenia and strain (GRBAS).^31^ The patient’s perception of the impact of his or her voice disorder upon speech intelligibility was self-evaluated by a six-point scale using the Voice Handicap Index (VHI), which comprises functional (VHI-F), emotional (VHI-E) and physical (VHI-P) subscales,^32^ and using a speech Visual Analogue Scale (VAS) composed of voice quality (VAS 1–4), intelligibility (VAS 5–7) and pragmatics (VAS 8–9) subscales^33^ (Supplementary Appendix 2).

Prior to surgery, UPDRS motor scores were evaluated during ON and OFF states. Neuropsychological and voice/speech were evaluated during ON states only. OFF states followed 12 h (overnight) of drug discontinuation, and ON states were assessed 1 h after drug re-administration.

The levodopa equivalent daily dose (LEDD) was calculated as described by Tomlinson *et al*.^34^

### Planning of DBS trajectories

On the morning of the surgery, a Leksell frame was fixed to the patient’s head under local anaesthesia. Coordinates and trajectories were calculated using the iPlan stereotactic planning software (Brainlab) based on MRI. Shallow propofol sedation and local anaesthesia were given during burr hole drilling and fixation using Stimloc (Medtronic). MER and neurological testing were done under awake condition up to the implantation of the permanent electrodes (3389, Medtronic). Postoperative computed tomography (CT) was used for confirmation of leads’ locations and for ruling out any complications.

### Microelectrode recordings

For MER, the dura was coagulated and opened, and an Alpha Omega MicroGuide was introduced up to 25 mm from the MER target.^21,22^ Central and anterior polyamide-coated tungsten microelectrodes (2 mm apart) were introduced into a BenGun multielectrode holder. Impedances were in the range of 350–750 MΩ (measured at 1000 Hz). The MER was amplified by 10,000 or 25,000, band-passed from 250 to 6000 Hz using a hardware four-pole Butterworth filter. It was sampled at 24 or 48 kHz by a 12-bit A/D converter (using ±5 V input range). Discrete steps of 300–500 µm up to the dorsal border of the STN were used, which were further reduced to 50–100 µm until the ventral STN border. At each depth, about 5–10 s elapsed until the signal stabilized. MER was then conducted for about 30–60 s.

### Post STN-DBS outcomes

Following the DBS procedure, the neurologist chose the effective contact according to the best clinical response to stimulation and adjusted the stimulation and therapeutic parameters. The stereotactic coordinates of these active contacts were superimposed on the MER analyses. Postoperatively, all four possibilities (ON/OFF drugs * ON/OFF-DBS) were compared between pre- and post-STN-DBS. The ([ON-DBS/ON-drugs] − [OFF-DBS/ON-drugs]) seemed to be the most clinically relevant for two reasons: (i) patients used drugs both before and after surgery albeit at different dosages and (ii) both neuropsychological and voice scores are conventionally evaluated during the best performing time only, i.e. during ON periods.

### Statistical analysis

Univariate analysis (MEANS procedure) was applied for continuous variables and chi-squared test (FREQ procedure) for categorical variables. For isolating the impact of drugs from STN-DBS and their concomitant effect, we included variances and co-variances in the general linear model (GLM) plot (MIXED procedure). *R*^2^ selection methods (R-SQUARE( were used for correlating clinical scores with MER properties. Since both the neurophysiological and surgical values were retrieved intraoperatively, *R*^2^ was limited to subsets of one to three independent variables (for facilitating the decision-making procedure) that were correlated best with outcomes by linear regression with necessary corrections for multiple regressions.

Univariate analysis was performed to identify which neurophysiologic features of the STN were correlated with the MER trajectories and with clinical symptoms. Three STN features that were significantly correlated with the univariate outcomes were (i) the normalized root mean square (NRMS) of the neuronal activity; (ii) the STN width in millimetres; and (iii) the fraction of β-oscillations out of all STN length (frqβ/STN) together comprised the STN-DBS-MER score. These features were determined as described in Moran *et al.*^35^ To calculate the score, each of the three parameters comprising the score was divided into three sub-scores on a scale of 1–3: NRMS [(1) *X* < 1.5, (2) 1.5 < *X* < 2.5, and (3) *X* > 2.5]; STN width [(1) *X* < 3.5, (2) 3.5 < *X* < 5.5, and (3) *X* < 3.5 mm]; and frqβ/STN [(1) *X* < 1/3, (2) 1/3 < *X* < 2/3, (3) *X* > 2/3]. The final STN-DBS-MER score is a simple sum of the sub-score of each of the three parameters.

PC analysis (PCA) of motor, neuropsychological, voice and speech scores was performed, and Cronbach’s alpha (Cα) reproducibility of the PCs evaluated by their calculation in all four possible states (ON/OFF-Drug/DBS). For each of the six PCs, we chose its best representative by using Cα with a threshold of >0.6. To further test the reliability of the six PCs, we compared the correlation between each of the MER properties and the conventional clinical scores with the correlation between MER properties and the six PCs.

## Results

### Baseline characteristics of the study population

A total of 48 patients (19 women, 29 men) with idiopathic Parkinson’s disease (mean duration from diagnosis, 10.5 ± 4.3 years) were included in the study. The mean age at surgery was 58 years, and most patients (46/48, 95.8%) were right handed. Mean LEDD at baseline was 1225 ± 611 mg. Motor, neuropsychological and speech evaluations were done at a mean of 2.9 ± 0.77 months before surgery. Gender, age and duration of Parkinson’s disease did not affect the scores of these evaluations (Supplementary Table 1). Bilateral and unilateral DBS device implantation was performed in 27 and 21 patients, respectively. Among the patients with unilateral implantation, 13 and 8 were targeted to left and right STN, respectively. Bilateral DBS device implantation was done during the same surgery. No significant differences were found between the first and second implanted leads in bilateral cases.

### Patients’ pre-DBS profile

UPDRS-III (motor) scores showed a significant response to dopamine replacement therapy (mean 55%, range 37–65%). The OFF/ON drug scores were as follows: UPDRS-total (69.8/36.7, −52%), II (17.8/7.9, −55.6%) and III (44.4/20.9, 53%). UPDRS-I was 1.56, and UPDRS-IV was 6 ± 3.3 (mostly due to ON–OFF fluctuations). Mean S&E was 64.6 ± 16.6, and H&Y was 2.8 ± 0.6. UPDRS items were also grouped by symptoms and assessed during OFF/ON states: tremor (7/2, 71%), rigidity (9.3/4.1, 55%), limb bradykinesia (18/9.7, 46%) and axial signs (12/4.7, 59%). No significant differences were observed in the distribution of symptoms’ sub-types or severity across the cohort (Supplementary Table 1).

The majority of acoustic characteristics, speech intelligibility and neuropsychological scores were practically normal before DBS. Three VHI sub-scores were abnormally higher than 4 (*P* = 0.035): VHI-F 7.9, VHI-E 5.8 and VHI-P 7.2. The UPDRS-speech items (5 + 18) and swallowing items (6 + 7) mildly improved with dopamine replacement therapy (6.7/1.65 and 1.11/0.7, respectively; Supplementary Tables 1 and 2).

### Overall outcomes

Significant improvements in all motor symptoms (except axial signs) and LEDD reduction [a mean reduction of 38.5% (*P* = 0.046), from a mean baseline of 1225 ± 611 mg] were noted in all patients and independently of the laterality of DBS’s. Mild improvements were observed in posterior and mixed neuropsychological functions (verbal memory, visual memory and organization), in contrast with a mild deterioration in frontal functions (personality changes, executive functioning and verbal fluency). The concomitant decline in speech intelligibility was mild and only partial, probably in accordance with the neuropsychological verbal fluency results. Acoustic characteristics were least affected and remained within normal values.

A mean responsiveness of 55% to levodopa before surgery was common among better responders to DBS (Supplementary Table 1).

All patients and caregivers stated that the neuropsychological, voice and speech declines were tolerable compared to the significant overall improvement and reduction of ON–OFF fluctuations.

### Motor outcomes

**Figure 1:**
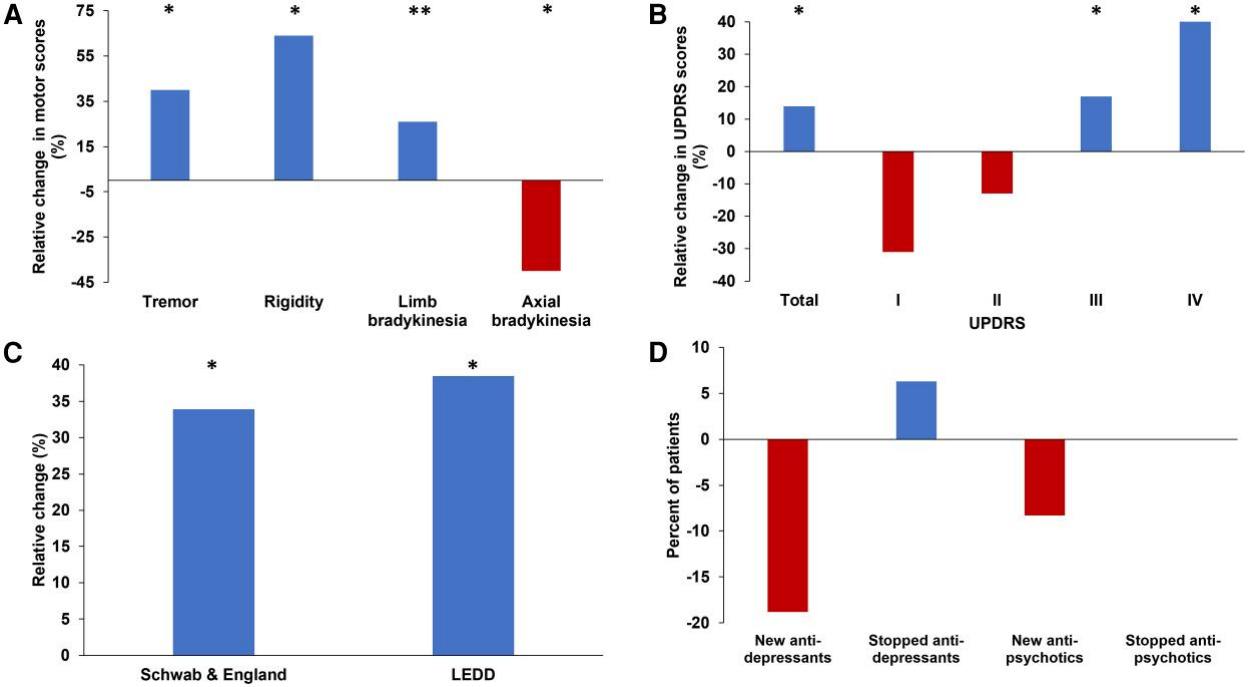
**Change in motor symptoms, ADL and medication postsurgery.** (**A**) All motor symptoms significantly improved (limb bradykinesia *P* = 0.0001; rigidity *P* = 0.006; tremor (*P* = 0.02), except axial signs, which persistently declined (*P* = 0.03). (**B**) UPDRS-total, UPDRS-III and UPDRS-IV significantly improved (*P* < 0.05, *P* = 0.0016 and *P* = 0.001, respectively) in contrast with UPDRS-I and UPDRS-II. (**C**) Both S&E score for ADL and LEDD significantly improved (*P* = 0.016 and *P* = 0.046, respectively). (**D**) A net increased usage of both anti-depressants and anti-psychotics was noted following STN-DBS. *P < 0.05; **P < 0.0001. Exact *P*-values are noted in the figure legend. DBS, deep brain stimulation; LEDD, levodopa equivalent daily dose; STN, subthalamic nucleus; UPDRS, unified Parkinson’s disease rating scale.

The interval from surgery to postoperative evaluation was 16.1 ± 1.4 months. Improvement was noted in limb bradykinesia (−2.5, −26%, *P* = 0.0001), rigidity (−1.66, −64%, *P* = 0.006) and tremor (−0.76, 40%, *P* = 0.02). Axial symptoms persisted and further deteriorated (2.5, 40%, *P* = 0.03; Fig. 1A). UPDRS-total significantly improved (−5.2, −14%, *P* < 0.05), UPDRS-I and UPDRS-II non-significantly worsened by 31% and 13%, respectively, while UPDRS-III (−3.7, −17%, *P* = 0.0016) and UPDRS-IV (−2.8, −46%, *P* = 0.001) significantly improved (Fig. 1B). Significant improvements were also noted in S&E ADL, which improved by 33.9%: from 64.6 (i.e. ‘some dependency’) before DBS device implantation to 86.56 (‘completely independent, aware of difficulties’) postsurgery (*P* = 0.016). LEDD decreased by 38.5% (*P* = 0.046; Fig. 1C). Post-DBS, 8.3 and 18.8% of patients needed new anti-psychotic and new anti-depressants, respectively, while 6.3% stopped taking anti-depressants. No patient stopped taking anti-psychotics (Fig. 1D).

### Neuropsychological outcomes

**Figure 2:**
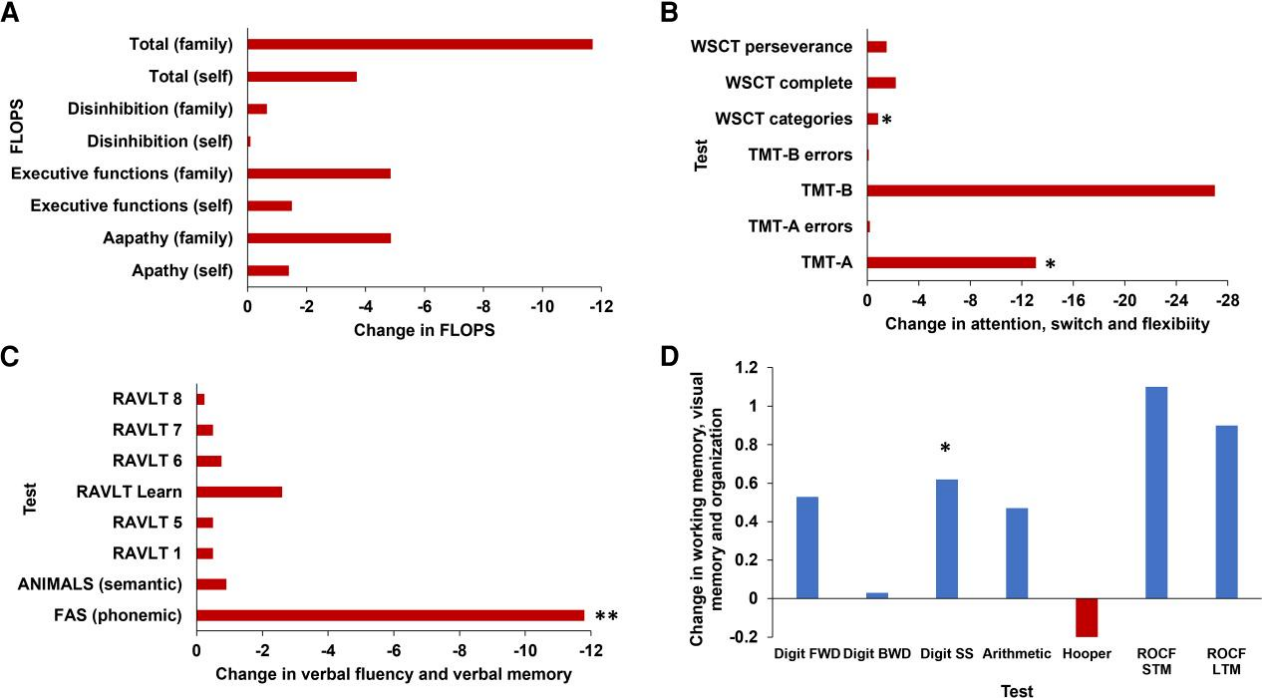
**Change in neuropsychological outcomes postsurgery.** (**A**) All frontal functions of FLOPS declined but were not statistically significantly. (**B**) Attention, attention switch and cognitive flexibility declined (WCST *P* = 0.0008; TMT-A *P* = 0.0072). (**C**) Among the verbal scores, the most significant decline was in the phonemic (frontal function—FAS) verbal fluency score (*P* < 0.0001) and less in the posterior function (ANIMALS) and verbal memory (RAVLT). In contrast, mixed functions such as working memory and posterior functions that relate to verbal memory (RAVLT Scores) (**C**) and visual memory (ROCF scores) (**D**) have improved to different degrees. *P < 0.05; **P < 0.0001. Exact *P*-values are noted in the figure legend. FAS, phonemic verbal fluency; FLOPS, Frontal Lobe Personality Scale; Digit BWD, Digit Span Backward; Digit FWD, Digit Span Forward; Digit SS, combined Forward/Backward Digit Span Score; LTM, long-term memory; RAVLT, Rey Auditory Verbal Learning Test; ROCF, Rey–Osterrieth Complex Figure Test; STM, short-term memory; TMT-A, Trail Making Test Part A; TMT-B, Trail Making Test Part B; WCST, Wisconsin Card Sorting Test.

Frontal neuropsychological functions declined while posterior and mixed functions partially improved. The three most significant deteriorations were noted in three frontal functions, as shown by the differences in measurements before and after surgery: (i) frontal lobe personality tests (FLOPS questionnaires: family-total 11.7, family-apathy 4.86 and family-executive function 4.85, *P* = 0.052–0.068); (ii) attention and executive functioning (WCST categories −0.82, *P* = 0.0008, TMT-A 13.10; *P* = 0.0072); and (iii) FAS (−11.8, *P* < 0.0001; Fig. 2A–C).

Mixed and posterior functions generally improved postsurgery. Working memory scores, which included frontal and posterior functions, have improved with different degrees of significance (Digit Span forward 0.53, combined forward and backward Digit Span 0.62, *P* = 0.03). Among the posterior verbal memory functions, only two out of six mildly declined (RAVLT6 −0.76, *P* = 0.05; RAVLTLearn −2.6, *P* = 0.08). None of the visual memory and perception tests (ROCF and Hooper) declined. Mild but non-statistically significant improvement was noted between the two sub-scores (ROCF STM 1.13, *P* = 0.28; ROCF LTM 0.9, *P* = 0.31; Fig. 2B–D; Supplementary Table 3).

### Speech and voice outcomes

**Figure 3:**
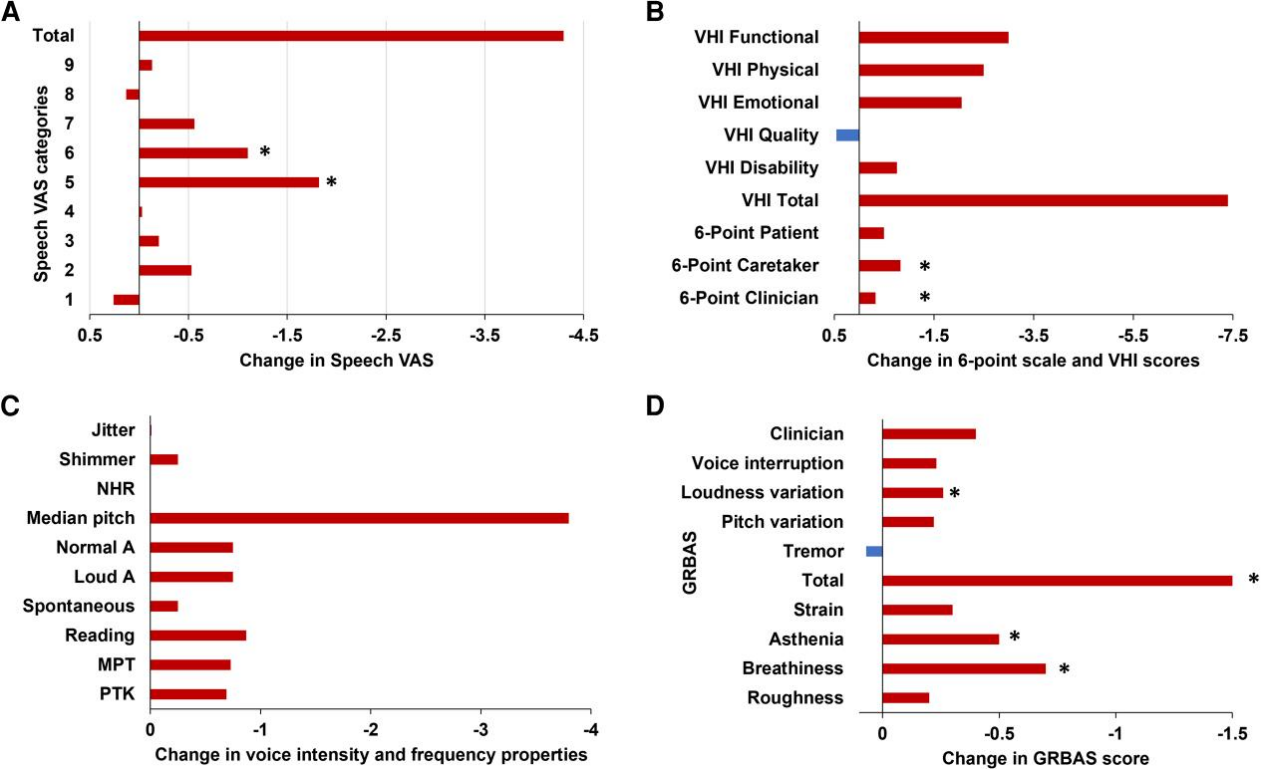
**Speech intelligibility outcomes postsurgery.** All speech and voice deteriorations were mild. (**A, B**) Among the speech intelligibility scores, VAS-5, VAS-6 and six-point scale significantly decreased (*P* < 0.001, *P* = 0.01 and *P* = 0.035, respectively). (**C, D**) Among the acoustic characteristics, only (**D**) GRBAS scores were most sensitive and significantly increased (GRBAS breathiness *P* = 0.005; asthenia *P* = 0.04; strain *P* = 0.06; total *P* = 0.03; loudness variation *P* = 0.03). Univariate analysis (MEANS procedure) was applied for continuous variables and chi-squared test (FREQ procedure) for categorical variables (*n* = 48 for all analyses). *P < 0.05. Exact *P*-values are noted in the figure legend. GRBAS, grade, roughness, breathiness, asthenia and strain; MPT, mean phonation time; NHR, noise to harmonic ratio, VAS, Visual Analogue Scale; VHI, Voice Handicap Index.

The vast majority of speech intelligibility and acoustic characteristics remained normal. Overall, acoustic characteristics’ scores were less significantly altered compared with speech intelligibility scores. Four speech intelligibility scores significantly deteriorated: VAS-5 (−1.82, *P* < 0.001), VAS-6 (−1.1, *P* = 0.01), UPDRS-speech (−0.7, *P* = 0.03) and six-point scale, clinician, caregiver and patient [−0.33 (*P* = 0.003), −0.83 (*P* = 0.01) and −0.56 (*P* = 0.06), respectively]. Among the physical properties of the voice, the same trend was noted for the PTK test (0.69, *P* = 0.05) and GRBAS sub-scores (GRBAS breathiness 0.6, *P* = 0.005; asthenia 0.7, *P* = 0.04; strain 0.3, *P* = 0.06; total 1.5, *P* = 0.03; loudness variation 0.26, *P* = 0.03). The change in the other speech scores did not reach statistical significance (Fig. 3A–D).

All mean speech physical properties remained within normal range following DBS device implantation. None of the acoustic analysis parameters (jitter, shimmer and NHR) significantly changed, and patients’ age or gender did not affect them. Although the mean median pitch remained within normal range with an overall non-significant change (−3.8, NS), analysis by gender showed a trend for statistical significance (*P* = 0.05) on the impact on median pitch (i.e. the fundamental frequency): while it decreased among 80% of the female patients [from 203 ± 38 to 189 ± 31 Hz (normal range: 140–220 Hz), *n* = 19], it was almost unchanged in the male patients [from 135.2 ± 23 to 134.5 ± 26 Hz (normal range: 85–180 Hz), *n* = 29] (Supplementary Fig. 1).

### The impact of non-motor deteriorations and the differences between patient’s self-assessments and caregivers’ assessments

Patients, caregivers and clinicians provided similar estimations of personality scores (FLOPS) and speech intelligibility six-point-scale score before DBS device implantation. However, postsurgery caregivers’ and clinicians’ scores presented a statistically significant worse change in these parameters (*P* = 0.01 and *P* = 0.003, respectively) compared to the patients’ self-estimation of outcome which did not significantly change (*P* = 0.6; Figs. 2A and 3B; Supplementary Table 4).

Notably, there were significant differences between patients’ and families’ FLOPS scores following STN-DBS. Scores given by families were always worse compared to the patient’s self-estimation for apathy (1.3, NS versus 4.86, *P* < 0.05), disinhibition (1.3, NS versus 4.85, *P* < 0.05), executive functioning (1.3, NS versus 4.86, *P* = 0.05) and total (3.7, NS versus 11.7, *P* < 0.05; Fig. 2A; Supplementary Tables 3 and 4).

### Identification of MER properties that correlate with conventional clinical scores

**Figure 4:**
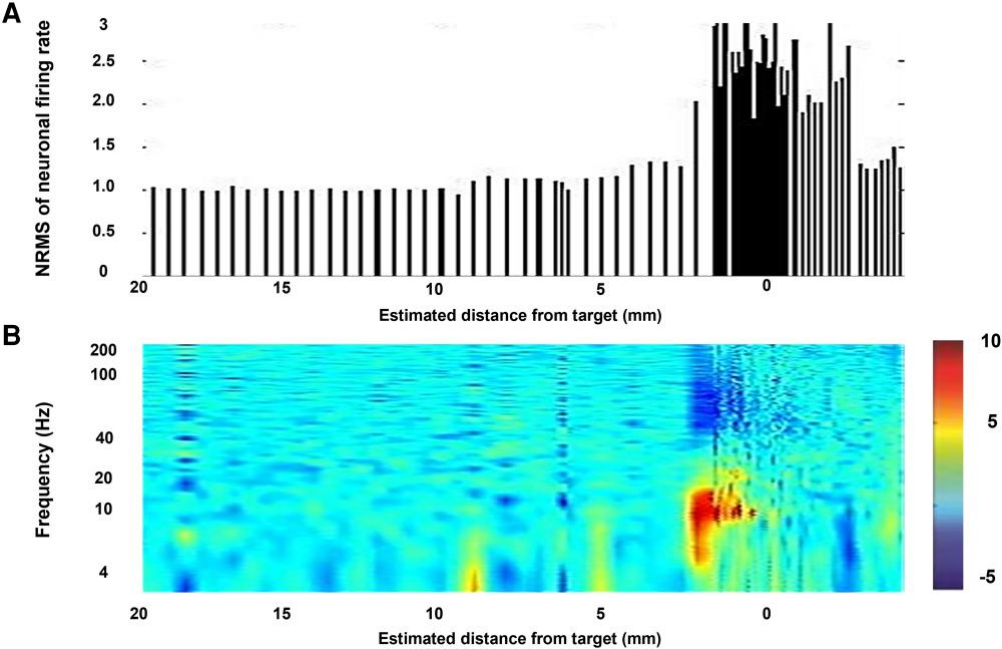
**The occurrence of β-oscillations across the cohort.** (**A**) NRMS of neuronal firing rate by estimated distance from the centre of the STN (0) based on presurgical MRI planification. MER exploration began 20 mm dorsal to this point. (**B**) A spectral analysis of the neuronal activity at 0–250 Hz by estimated distance from the centre of the STN. In each of the 198 trajectories, five MER properties of the STN were tracked: (i) NRMS of the neuronal firing rate; (ii) STN width (defined by the increased NRMS); (iii) DLOR width; (iv) DLOR/STN ratio; and (v) β-oscillation occurrence across the cohort. *x*-axes in both upper (**A**) and lower (**B**) figures describe the distance from the estimated centre of the STN (0) based on presurgical MRI planification. MER exploration began 20 mm dorsal to this point. (**B**) A spectral analysis of the neuronal activity was done at 0–250 Hz.

**Figure 5:**
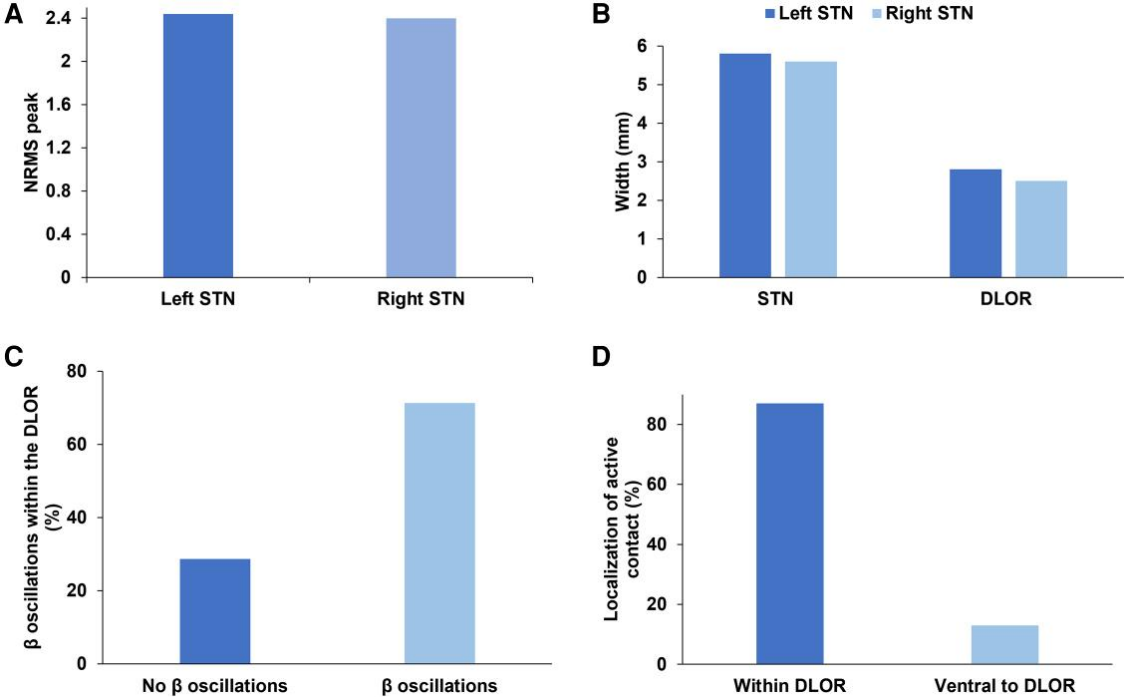
**MER properties of the STN.** (**A**) Mean NRMS-peak by STN side (overall mean was 2.44 ± 1.5). (**B**) Mean STN and DLOR widths by STN side. (**C**) β-Oscillations within the DLOR. In 71.3% of trajectories, a β-oscillatory increment was found in the dorsolateral STN, and 58% had a concomitant decreased γ-oscillatory activity. (**D**) Localization of the active contact in relation to the DLOR. Eighty-seven per cent of clinically active contacts were within the DLOR. DLOR, dorso-lateral oscillatory region; MER, microelectrode recordings; NRMS, normalized root mean square; STN, subthalamic nucleus.

Analyses of 198 MER trajectories identified three neurophysiologic features of the STN that significantly correlated with outcomes: (i) NRMS of the neuronal activity; (ii) the STN width; and (iii) frqβ/STN (Fig. 4A and B). The mean NRMS-peak was 2.44 ± 1.5, and the STN width was 5.58 ± 1.5 mm with a frqβ/STN of 44% (2.58 ± 1.4 mm; Fig. 5A and B). In 71.3% of the trajectories, a β-oscillatory increment was found in the dorsolateral STN (Fig. 5C), but no such oscillations were found in the ventral STN tier.

These three MER properties were significantly correlated with better outcomes: (i) wider STN (*P* < 0.001); (ii) higher NRMS (*P* = 0.03); and (iii) frqβ/STN (*P* = 0.02). MER scores were correlated with better motor outcomes (except for axial signs) and less neuropsychological decline. Speech intelligibility showed a lower correlation, while acoustic characteristics were not correlated with these MER properties. Notably, 87% of clinically effective contacts—as defined by the neurologist once the patients achieved steady state with best motor outcomes and no neuropsychological/speech deficits—resided within the DLOR (Fig. 5D).

**Figure 6:**
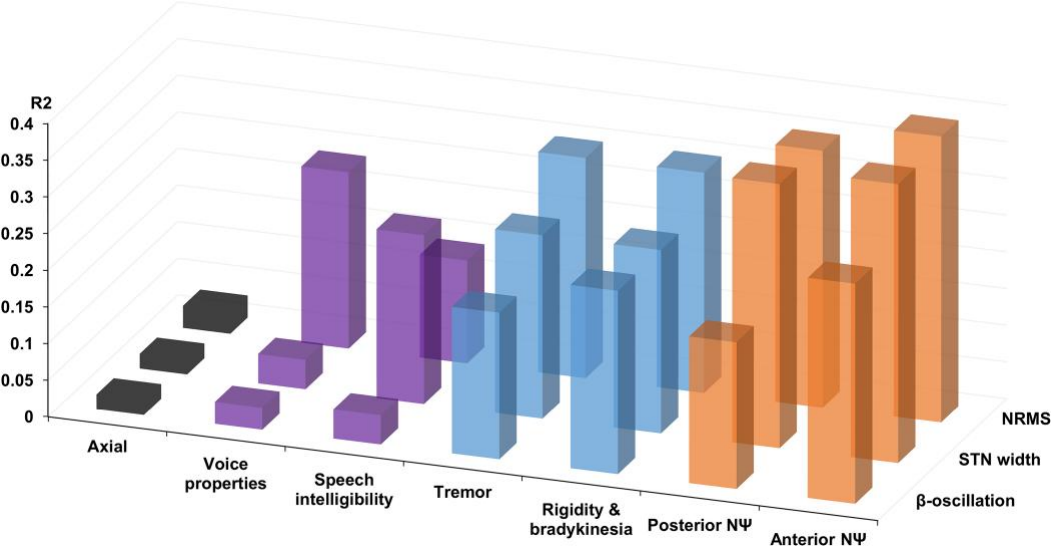
**MERs have distinctive predictive values on motor and neuropsychological scores compared to voice/speech.** All the symptoms (axial, acoustic characteristics, speech intelligibility, tremor, rigidity and limb bradykinesia, frontal and posterior neuropsychological symptoms) are aligned along the *x*-axes. *R*^2^ on the *y*-axes is presented regarding each of the MER properties (NRMS, STN width and β-oscillations). Univariate analyses (*N* = 48) show that NRMS and STN width have higher correlations compared with β-oscillations. However, these correlations differ regarding the different symptoms. Both NRMS and STN width highly (and similarly) correlate with motor (except axial signs) and neuropsychological scores compared with acoustics and speech. β-Oscillations correlate only with motor (except axial signs) and neuropsychological functions. Axial symptoms are the only ones not to significantly correlate with any of the MER properties. MER, microelectrode recordings; NRMS, normalized root mean square; STN, subthalamic nucleus.

Univariate analyses showed that NRMS and STN width are highly correlated with outcomes compared with frqβ/STN. However, these correlations differed by symptoms: both NRMS and STN width were highly (and similarly) correlated with motor and neuropsychological scores compared with voice and speech scores. frqβ/STN correlated only with motor and neuropsychological functions. Axial symptoms are the only variables that showed no correlation with any of the MER properties in univariate and multivariate analyses (Fig. 6).

MER properties that showed no correlation with any of the outcomes included a concomitant decreased γ-oscillatory activity in the DLOR (found in 58% of the 198 MERs) and scattered theta–delta oscillations typically 5–10 mm dorsal to the STN entry.^35^ The mean coordinates referring to the mid-commissural point—lateral (*x*: 10.33 ± 0.72), anteroposterior (*y*: −2.59 ± 0.55) and vertical (*z*: −4.56 ± 0.85)—showed no significant differences among patients or between left and right STNs (Supplementary Table 5). The stimulation parameters were assessed twice during steady-state visits and did not show any significant variability (130–185 Hz, <4 V and 60–200 μs). Their values are in accordance with previously reported settings.^36,37^

### PCA of clinical scores

**Figure 7:**
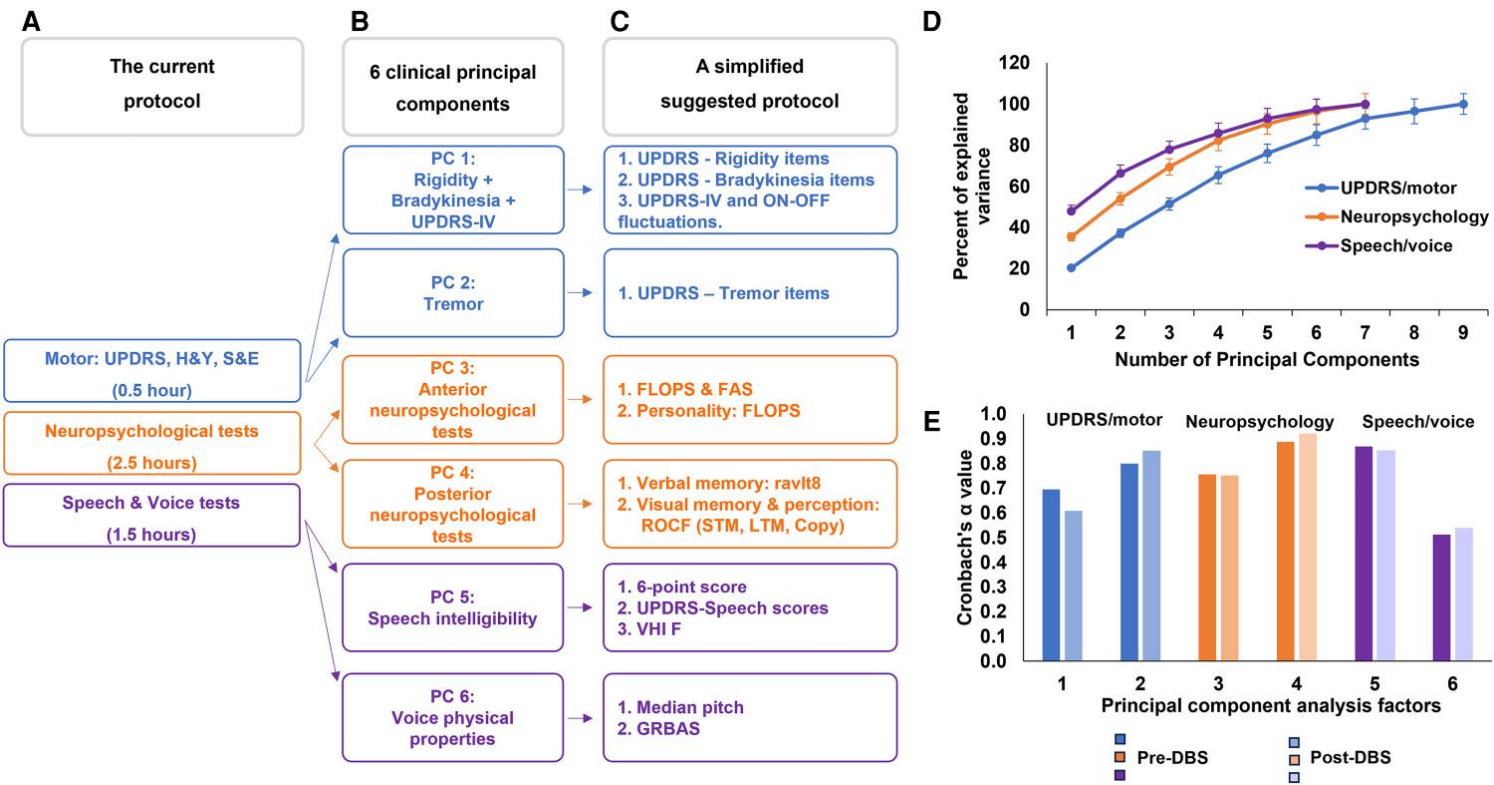
**A PCA-based simplified and thorough clinical scoring protocol.** (**A**) The actual clinical protocol (of >100 scores) takes more than 4.5 h and therefore requires two sessions to avoid ON/OFF transitions, which might distort results. (**B**) PCA of these motor, neuropsychological and acoustic/speech scores rendered six PCs, i.e. PC 1–PC 6. (**C**) Cronbach’s *α* (higher than 0.7, except for factor 6) renders a reliable representation of each of these six PCs, which simplifies the protocol into a single session of not >1.5 h. (**D**) The explained variance is presented for each of the symptoms’ sub-groups (motor, neuropsychological and acoustic/speech) with error bars representing the differences between pre- and post-DBS calculations. (**E**) Cronbach’s *α* values of the selected tests are presented, showing similar values before and after STN-DBS (*N* = 117). DBS, deep brain stimulation; FAS, phonemic verbal fluency; FLOPS, Frontal Lobe Personality Scale; GRBAS, grade, roughness, breathiness, asthenia and strain; H&Y, Hoehn and Yahr; LTM, long-term memory; PC, principal component; RAVLT, Rey Auditory Verbal Learning Test; ROCF, Rey–Osterrieth Complex Figure Test; S&E, Schwab and England; STM, short-term memory; UPDRS, unified Parkinson’s disease rating scale; VHI, Voice Handicap Index.

PCA of more than 100 scores (motor, neuropsychological, voice and speech) yielded six independent PCs: PC 1: bradykinesia, rigidity and UPDRS-IV; PC 2: tremor; PC 3: frontal neuropsychological scores; PC 4: posterior neuropsychological scores; PC 5: speech intelligibility scores; and PC 6: physical properties of the voice. The variabilities among these factors and between pre- and post-DBS states were not statistically significant, advocating their reproducibility (Fig. 7E).

These six PCs reached an explained variance of 60% of the clinical scores. On the neuropsychological level, the PCs well differentiated the frontal (executive functioning and personality traits) from the posterior (language and memory) neuropsychological functions. Last, acoustic characteristics were well differentiated from speech intelligibility (Fig. 7D). For each of the six PCs, we chose its best representative by using Cα with a threshold >0.6. Most of the neuropsychological, voice and speech scores were found to be redundant. Frontal neuropsychological outcomes (PC 3) were best represented by FLOPS and FAS scores. Posterior neuropsychological outcomes (PC 4) were best represented by ROCF ± RAVLT8 (Cα > 0.75). For speech intelligibility (PC 5), the six-point scale together with UPDRS speech items and VHI-F suffice (Cα > 0.85). Acoustic characteristics (PC 6) were best represented by GRBAS and median pitch (Cα = 0.52). The last was chosen due to the gender differences and despite the mean non-significant changes following DBS (Supplementary Fig. 1).

Thus, using this approach, we propose a simplified combined motor, neuropsychological, voice and speech protocol. The proposed protocol excludes redundant scores and simplifies the 4.5-h-long clinical evaluation (together with consultations; Fig. 7A) to a 1.5-h one (Fig. 7C), reducing the risk for ON–OFF fluctuations during the evaluation (Supplementary Table 6).

### Correlation of the integrative STN-DBS-MER score with PCA results

**Figure 8:**
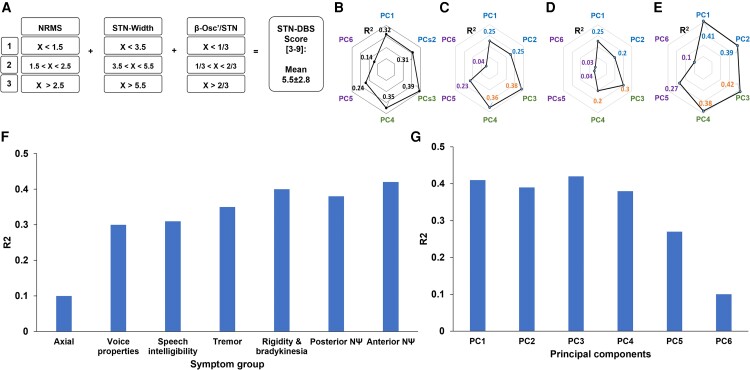
**Predictive values of STN-DBS-MER score on outcomes.** (**A**) Each sub-score (NRMS, STN width and β-oscillation/STN) is graded between 1 and 3. The STN-DBS score is a simple linear summation of each of the three sub-scores (range 3–9). The mean STN-DBS score was 5.5 ± 2.8. (**B–D**) Each of the three MER sub-scores shows a significant correlation with all symptom sub-groups, except axial signs, and a non-significant correlation with acoustic characteristics. (**E**) The STN-DBS score, which integrates these MER sub-scores, maintains these patterns. Higher STN-DBS scores indicate better motor outcomes (except axial signs) and fewer neuropsychological and speech adverse effects (except acoustic characteristics). (**F**) Correlation between STN-DBS score and symptom groups. (**G**) Correlation between STN-DBS score and PCs. STN-DBS score similarly correlates with the symptom groups and the PCs. *R*^2^ selection methods (R-SQUARE) were used for predicting clinical scores by STN-DBS scores (*N* = 48 for all). DBS, deep brain stimulation; MER, microelectrode recordings; NRMS, normalized root mean square; PC, principal component; STN, subthalamic nucleus.

The STN-DBS-MER score ranged from 3 to 9 with a mean of 5.5 ± 2.8 (Fig. 8A).

To further test the reliability of the six PCs, we compared the correlation between each of the MER properties and the conventional clinical scores with the correlation between MER properties and the six PCs. This comparison showed no statistically significant differences further advocating the usage of the PCA results (Fig. 8B–E).

The pattern that emerged from the univariate regressions between each of the STN-DBS constituents and the conventional clinical scores was maintained and reproduced twice: first, it was reproduced by the multivariate regression between the STN-DBS score and the conventional clinical scores, suggesting that this score does not alter the correlations with outcomes (Fig. 8F compared to Fig. 7). Second, similar patterns were found in relation to the six PCs—both in relation to the STN-DBS score and for each of its constituents (Fig. 8G).

Higher STN-DBS scores indicated better motor outcomes (*R*^2^ = 0.4) except for axial signs. A similar regression score (*R*^2^ = 0.4) was found for neuropsychological outcomes. While speech intelligibility (PC 5) maintained a significant regression score (*R*^2^ > 0.3), acoustic characteristics (PC 6) showed a weak regression (*R*^2^ = 0.14). None of the MER properties or the combined STN-DBS score was correlated with axial outcomes (Fig. 8E).

## Discussion

Overall, all patients significantly improved during long-term follow-up. Both patients and caregivers reported acceptable neuropsychological and speech declines. Our objective was to find markers that can reduce the risks for neuropsychological and speech declines while optimizing motor outcomes.

We have designed an innovative algorithm that assists in deciding among optional trajectories in terms of long-term (1–1.5 years) motor and non-motor outcomes. The suggested STN-DBS score is a simple summation of three MER properties: NRMS, STN width and the frqβ/STN. Each of them received a score ranging from 0 to 3. A total STN-DBS score higher than 5.5 (typically composed of NRMS > 1.5, an STN width > 3 mm and a frqβ/STN > 1/3) was correlated with improved motor outcomes and less non-motor complications. The 2-mm STN width parameter was chosen in order to simulate the interval between adjacent contacts of commonly used electrodes with 1.5 mm length contact and 0.5 mm gap between contacts. For example, a 4-mm-wide STN should contain approximately two contacts.

The differences between family/caregivers’ postoperative assessments and patients’ self-assessments are notable. Both personality and speech intelligibility scores showed that patients perceived their post-STN-DBS status better than did their families/caregivers. Moreover, although a trend only, shorter idiopathic Parkinson’s disease duration (8–9 years versus 12–13 years) indicated improved ADL, ON/OFF fluctuations, UPDRS-IV and absolute motor scores (Supplementary Table 4). This finding is in accordance with the results of the ‘EarlyStim’ study.^38-41^

### Why do most effective contacts reside within the DLOR?

Division of the STN into thirds was based on the three functional territories of the STN.^39-41^ All motor symptoms, except axial signs, significantly improved and remained stable. Recent studies have emphasized this issue, identifying a kinetic rigid patient as a unique subgroup of idiopathic Parkinson’s disease. Our findings, which grouped these symptoms to distinct PCs (both before and after DBS device implantation) and their unique refractoriness to STN-DBS, seem in accordance with these recently suggested clinical stratifications.

The overall mild deterioration in executive performances (frontal neuropsychological functions) was previously explained by the possible spread of the STN-DBS to non-motor parts of the STN and/or the natural progression of the disease.^42^ This explanation seems to be in accordance with our findings that 87% of the clinically effective contacts were within the DLOR (either with or without β-oscillations) and were associated with less neuropsychological complications. Our results showed improvements in working memory. These were previously reported and explained by the ‘releasing the brake’ model, which suggests that STN-DBS reduces the basal ganglia inhibition of the frontal cortex and therefore improves these frontal properties.^39,43^

Physical properties of voice and speech intelligibility mildly deteriorated following STN-DBS. These categories differ in their relation with MER and their overall alteration post-STN-DBS. Most of the scores changed non-significantly, keeping both voice and speech scores within normal range. Despite the overall lower correlation between MER and speech and voice, speech intelligibility scores were more sensitive. Among these, VAS, GRBAS and the six-point scale should be maintained when evaluating speech post-STN-DBS. Concerning the physical properties of the voice, none of the acoustic analyses significantly changed postsurgery; however, the mean median pitch should be analysed by gender since it significantly changed in women only.

### Limitations

The limitations of our study include its retrospective design and the limited cohort size of 48 patients. However, the relatively high number of MER trajectories (198), the correlation of their neurophysiological features with more than 100 clinical scores evaluated before DBS and the long postoperative follow-up (44 months postsurgery) provide additional weight to our conclusions. Despite the rich literature on DBS, a detailed analysis of the correlation between MER and a combined motor, neuropsychological and voice/speech outcome has not been presented yet. The present analysis may therefore contribute to the clinical management of these patients.

### Future perspectives

An important question deserving attention is which factors/scores correlate with the patients’ own perception of Parkinson’s disease severity? Is it the absolute UPDRS-motor score or the degree of ON/OFF fluctuations? This question is important because it relates to the reasons for which the patient considers DBS. The alternative interpretation of the conventional UPDRS through ‘Symptom Scores’ seems suitable for this purpose. In our cohort, ADL significantly correlated only with the UPDRS-IV. These findings suggested that the perception of ADL performance by patients with idiopathic Parkinson’s disease might be mainly affected by ON/OFF/UPDRS-IV rather than by the absolute severity of symptoms. These observations prompt us to look for the main factor affecting symptom fluctuation (part of UPDRS-IV). Reduction in fluctuations should be a major aim of surgery, and its correlation with MER properties should be further studied.

## Conclusion

Neurophysiological properties derived during intraoperative MER are correlated with all motor outcomes (except axial signs), frontal and posterior neuropsychological functions and, to a limited extent, speech intelligibility (but not physical properties of the voice). We suggest a simple STN-DBS score, based on a summation of three intraoperative MER sub-scores: NRMS, STN width and the relative DLOR proportion, to assist neurosurgeons, neurologists and neurophysiologists in defining the best target for permanent DBS of the STN among patients with Parkinson’s disease and predicting favourable motor outcomes and less non-motor adverse effects that can be thoroughly evaluated using a PCA-based simplified scoring protocol. Our findings should be validated under controlled conditions, especially regarding the magnitude of reduction of concomitant therapy. If validated, the suggested algorithm can be used for the prediction of DBS outcomes.

## Supplementary Material

fcad268_Supplementary_Data

## Data Availability

The data that support the findings of this study are available upon request from the corresponding author. The data are not publicly available because they comprise information that could compromise the privacy of research participants.
