# Supplementary material for: Subthalamic nucleus physiology is correlated with deep brain stimulation motor and non-motor outcomes
Source: Brain Commun. 2023 Oct 18;5(6):fcad268. doi: 10.1093/braincomms/fcad268 (PMC10664412; doi:10.1093/braincomms/fcad268)
Supplement: fcad268_Supplementary_Data [file fcad268_supplementary_data.pdf]

SUPPLEMENTARY DATA

Supplementary Appendix 1. Detailed neuropsychological scores

Supplementary Appendix 2. Detailed voice and speech scores

Supplementary Table 1. Pre-STN-DBS clinical, UPDRS, motor and LED scores

Supplementary Table 2. Pre-STN-DBS voice and speech scores

Supplementary Table 3. Pre-STN-DBS neuropsychological scores

Supplementary Table 4. Summary of pre- and post-STN-DBS clinical scores

Supplementary Table 5. Stereotactic properties and MER properties

Supplementary Table 6. Practical recommendation for using the STN-DBS score

Supplementary Figure 1. Median pitch is the only score that changes by gender.

Supplementary Appendix 1. Detailed neuropsychological scores

Conventional tools were used before and after surgery to evaluate 7 neuropsychological faculties as described below. The differentiation between anterior and posterior properties are noted.[1]

Anterior neuropsychological scores

Anterior lobe functions were evaluated with the Frontal Lobe Personality Scale (FLOPS) which covers cognitive and behavioural manifestations associated with anterior lobe functioning and the integrity of anterior-subcortical circuits. It yields 3 subscale scores: apathy (FLOPSsselfa, FLOPSsfama), disinhibition (FLOPSsselfd, FLOPSsfamd), and executive function (FLOPSsselfe, FLOPSsfame), as well as a total score. In addition, the Anterior Lobe Family Evaluation Scale was administered to one family member of each patient to provide an additional perspective.

Attention and working memory were evaluated with the Digit Span Forward (DGSfwd), Digit Span Backward (DGSbwd), combined forward/backward score (ssDGS) and verbal subtests of the Wechsler Adult Intelligence Scale (WAIS)-III. Working Memory was also evaluated with the Arithmetic WAIS-III subtest.[2] The Trail-Making Test Part A (TMT-A)[3] was used to evaluate simple attention functioning and processing speed.

Attention shifting and cognitive flexibility were evaluated with the Trail-Making Test Part B (TMT-B). In addition, the Wisconsin Card Sorting Test (WCST) was used to evaluate executive functions, including problem-solving skills and cognitive flexibility ("set shifting" - wiscat, wicsom, wispersv).[3]

Phonemic verbal fluency (an anterior faculty) was examined by a non-formal Hebrew 3-letter phonemic fluency task (FAS) and a one-category semantic fluency task (ANIMALS – a posterior faculty) developed at the Hebrew University of Jerusalem.[4]

Posterior neuropsychological scores

Visuospatial functions and organization were evaluated with the Rey-Osterrieth Complex Figure copy test (ROCFcopy) and the Hooper test (hooper), respectively.[5] In addition, Digit Symbol (digitsym), a subtest of the WAIS-III, was used to evaluate visuospatial functioning and processing speed.[2]

Immediate and delayed Visual Memory were evaluated with ROCF-STM (short-term memory) and ROCF-LTM (long-term memory), respectively.[5]

Verbal learning and memory were evaluated with the Hebrew version of the Rey Auditory Verbal Learning Test (RAVLT).[6] The test includes verbal information encoding, verbal proactive interference (RAVLT6), immediate and delayed verbal memory recall, and verbal learning (RAVLTlearn).

Supplementary Appendix 2. Detailed voice and speech scores

Physical properties of the voice

Acoustic characteristics of voice parameters of sustained vowels /a/, repetitions of /pataka/, and sentence production were evaluated with the PRAAT voice analysis program.[7] as follows. All of these parameters are gender-independent.

Fundamental Frequency (~*Median Pitch)* is a measure of the number of cycles the vocal folds could make in one second. Normal ranges differ between men (85-180 Hz) and women (165-255 Hz).

Jitter is a measure of the variability or perturbation of the fundamental frequency. It is affected mainly by lack of control of vocal-fold vibrations. Values range from 0 to 200%, with >1.04% considered pathological. Higher scores indicate worse clinical status.

Shimmer is the variability or perturbation of the amplitude of the sound wave or intensity of the vocal emission. It is affected by a reduction of glottic resistance and mass lesions in the vocal folds. Values >3.08% are considered pathological. Higher scores indicate worse clinical status.

Harmonics-to-Noise-Ratio defines the degree of acoustic periodicity. It is determined by calculating the energy of the signal and noise [10*$\log_{10}$ (harmonics/noise)]. A value of 0 means there is equal energy in the harmonics and the noise. No threshold exists. Higher ratios indicate worse status.

Diadochokinetic Rate is a measure of the structural and physiological changes in the central nervous system and the peripheral components of the oral and speech production mechanism. It is evaluated by /PTK/ repetition. The normal rate in adults is PTK*10 ≤5sec.

Maximal Phonation Time (MPT) is a measure of glottic efficiency, that is, the ability to close (adduct) the vocal folds efficiently and easily and to vibrate them strongly through rapid opening and closing cycles. It determines the maximum time (in seconds) a person can sustain a vowel sound (/ah/, /ee/, /oo/) on a single deep breath at a relatively comfortable pitch and loudness. The final score is the best score of 3 ‘ah’ attempts. The normal MPT range is 25-35 seconds in adult males and 15-25 seconds in adult females. A time of <7 seconds is definitively pathological.

Speech intensity is evaluated using a computerized acoustic analysis of the intensity of normal /a/ (normal ~ 60 dB), loud /a/ (normal ~ 90 dB), spontaneous speech (normal ~ none), and reading (normal ~ none).

Voice Quality is evaluated with the GRBAS scale, a widely used perceptual instrument[8] that covers 5 parameters: grade (of hoarseness), roughness, breathiness, asthenia, and strain. Each parameter is graded on a scale of 0 to 3. The instrument is completed by trained clinical staff. The final score indicates the severity of dysphonia. The f GRBAS is also used to evaluate the presence (yes/no) of tremor/pitch variation/loudness variation/voice interruption/other.[9]

Speech intelligibility

Patient, caregiver, and clinician perceptions of speech intelligibility were evaluated with the Voice Handicap Index (VHI), Speech Visual Analog Scale (VAS), 6-Point Scale, and UPDRS-Speech subscale.[8]

The *VHI* assesses self-perceived impact/interference of an individual's "voice disorder" on the social aspects of his or her life. It contains 30 statements relating to functional (VHI-F), physical (VHI-P), and emotional (VHI-E) issues of a voice disorder. Each is scored from 0 to 4. In addition, the VHI includes 2 independent items on the degree of disturbance rated from 1 to 7 and the quantity of communication/speech during the day (rated from 7 to 1).

The *Speech VAS* is a nine-item form completed independently by the patient's caregiver and clinician to evaluate voice quality (item 1-4), intelligibility (items 5-7), and pragmatics (items 8, 9). All are scored on a scale of 1 (normal) to 10. These scales can be rearranged and grouped to assess functionality as follows: Voice Properties = VAS1-4 (+ VHI/P); Speech Intelligibility = VAS 5-7 (+ VHI/F+E); Pragmatic Function = VAS 8-9 (+ VHI/F+E)

The 6-Point-Scale is a one-item instrument measuring speech intelligibility intended to be completed independently by the patient, the patient's caregiver, and the clinician. The responder is asked to select which statement out of 6 best describes the patient's current status (6 is normal).

The UPDRS-Speech subscale comprises UPDRS items 5+18.[10]

References

1. Miller IN, Neargarder S, Risi MM, Cronin-Golomb A. Frontal and posterior subtypes of neuropsychological deficit in Parkinson's disease. *Behav Neurosci.* 2013; **127**(2): 175-183.
2. Wechsler D. A Standardized Memory Scale for Clinical Use. *The Journal of Psychology.* 1945; **19**(1): 87-95.
3. Reitan RM. The relation of the trail making test to organic brain damage. *J Consult Psychol.* 1955; **19**(5): 393-394.
4. Kavé G. Phonemic fluency, semantic fluency, and difference scores: normative data for adult Hebrew speakers. *J Clin Exp Neuropsychol.* 2005; **27**(6): 690-699.
5. Shin MS, Park SY, Park SR, Seol SH, Kwon JS. Clinical and empirical applications of the Rey-Osterrieth Complex Figure Test. *Nat Protoc.* 2006; **1**(2): 892-899.
6. Vakil E, Blachstein H. Rey AVLT: Developmental norms for adults and the sensitivity of different memory measures to age. *The Clinical Neuropsychologist.* 1997; **11**(4): 356-369.
7. *Praat: doing phonetics by computer, version 4.4.24* [computer program]. 2004.
8. Midi I, Dogan M, Koseoglu M, Can G, Sehitoglu MA, Gunal DI. Voice abnormalities and their relation with motor dysfunction in Parkinson's disease. *Acta Neurol Scand.* 2008; **117**(1): 26-34.
9. Tanaka Y, Tsuboi T, Watanabe H, et al. Voice features of Parkinson's disease patients with subthalamic nucleus deep brain stimulation. *J Neurol.* 2015; **262**(5): 1173-1181.
10. Chun KA, Manley GT, Stiver SI, et al. Interobserver variability in the assessment of CT imaging features of traumatic brain injury. *J Neurotrauma.* 2010; **27**(2): 325-330.

Supplementary Table 1. Pre-STN-DBS Epidemiological, UPDRS, Motor and LED scores

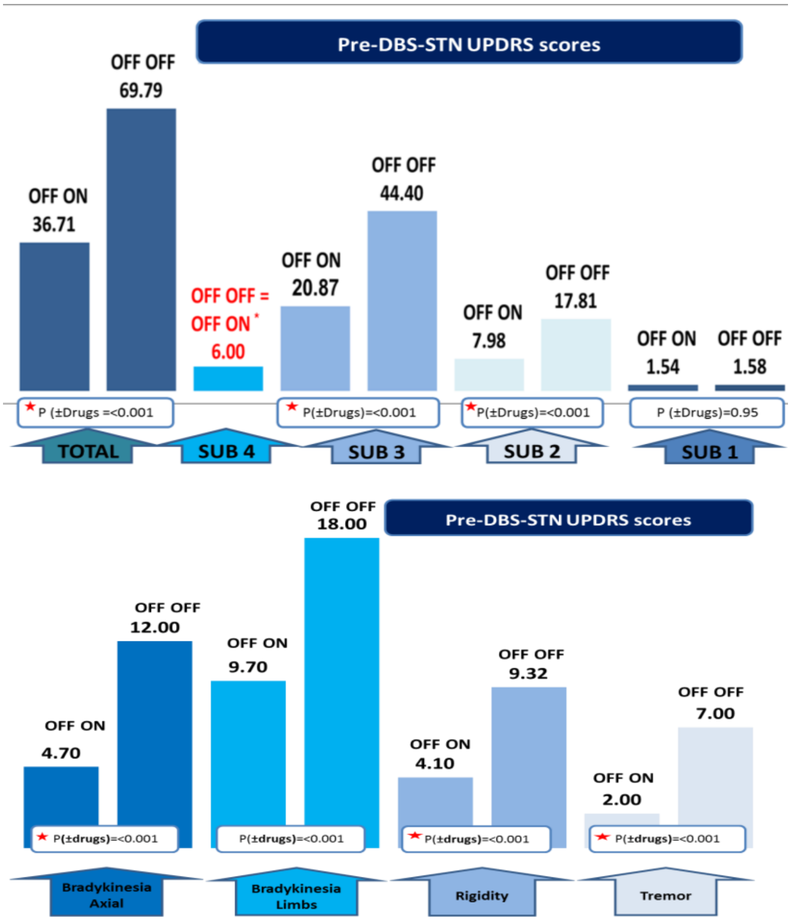

Epidemiological parameter	Mean ± SD
Age at STN-DBS	58±8 years
Gender	19 Females, 29 Males
Duration of Parkinson's disease	10.5±4.3
LEDD	1225±611
Schwab & England scale score	64±16
Hoehn & Yahr scale score	2.8±0.6
Interval between Pre-DBS scoring and surgery	2.9±0.77 months
Hand dominance	46/48 Right
Mean LEDD responsiveness	55%

The distribution of motor symptoms (their type and severity) did not differ across the cohort, neither was the interval for scoring before DBS. A good response to LEDD is demonstrated. Schwab & England is a measure of activities of daily living.

Abbreviations: DBS, deep-brain stimulation; LEDD: levodopa equivalent daily dose; SD, standard deviation; STN, subthalamic nucleus; UPDRS: unified Parkinson's disease rating scale

Supplementary Table 2. Pre-STN-DBS voice & speech scores

Pre-DBS Speech & Voice Score		
**Clinical Score**	**Mean**	**Normal range**
**Acoustic Computerized Analyses of Voice Recordings**		
Median Pitch (MP)	165.9	Men:85-180Hz Woman:165-220Hz
Jitter (MP variability)	0.4	Normal <1.04%
Shimmer (dB variability)	1.9	Normal <3.08%
Harmonic to Noise ratio	21.3	No norms
Speech Intensities (dB)	62-79	60-90 dB
Maximum phonation time	15.4	Normal >7 sec
Diadochokinetic score	4.5	Normal < 5
**GRBAS: Voice Grade/Roughness/Breathiness/Asthenic/Strain**		
Voice variation	0.3	A perceptual scorning of patients' voices: Normal 0 - Pathological 3
Loudness variation	0.1	
Pitch variation	0.1	
Voice tremor	0.2	
Grade	0.63	
Roughness	0.40	
Breathiness	0.38	
Asthenic	0.43	
Strain	0.06	

Pre-DBS Speech & Voice Score		
**Clinical Score**	**Mean**	**Normal range**
**6 Points Scores : A patient/caregiver/clinician questioner**		
Patient/Clinician/Caregiver	5.3/5.3/5	Scale of 6 (normal) to 1 (abnormal)
**Voice Handicap Index (VHI) : A patient questioner.**		
VHI Total	21.3	Abnormal 30
VHI F: Functional properties	**7.9**	Scale of 0 (normal) to 7 (abnormal)
VHI E: Emotional properties	**5.8**	
VHI P: Physical properties	**7.2**	
VHI Speech Disturbance	2.4	Scale of 0 (normal) to 4 (abnormal)
VHI Speech Quantity	4.4	
**Speech Visual Analogue Scale (VAS):Caregiver/clinician questioner**		
VAS Total	28.3	
VAS Pragmatics (9-8)	3.6	Scale of 1 (normal) to 10 (abnormal)
VAS Intelligibility (7-5)	2.9	
[illegible]	[illegible]	

PreDBS UPDRS Speech(5+18) & Swallowing/Salivation (6+7)		
**Clinical Score**	**Off Drgus**	**On Drugs**
Speech (5+18)	2.69	1.65
Swallowing/Salivation (6+7)	1.11	0.70

Supplementary - Table 2: Except VHI F,E and P all voice and speech properties were normal pre STN-DBS. **(Abnormal values in red).**

Abbreviations: DBS, deep-brain stimulation; SD, standard deviation; STN, subthalamic nucleus; UPDRS, unified Parkinson's disease rating scale; VHI, voice handicap index; VAS, visual analogue scale

Supplementary Table 3. Pre-STN-DBS scores

Pre-DBS Anterior neuropsychological faculties		
**Clinical Score**	**Subscales**	**Values**
**Working Memory**		
Digit Span Tests	Digit Span combined backward/ forward (SS)	9.16
	Digit Span Forward (FWD)	5.70
	digitsym	6.35
**Attention shifting & Executive Functioning**		
Trail Making Tests (TMT)	TMT-A	53.60
	TMT-A error	0.04
	TMT-B	121.50
	TMT-B error	0.92
Wisconsin Card Sorting Test (WCST)	WSCT categories	3.94
	WSCT complete	14.47
	WSCT perseverance	7.14
**Frontal Lobe Personality Scales Assessed by the patient and family**		
Frontal Lobe Personality Scales (FLOPS)	Apathy (self)	25.40
	Disinhibition (self)	23.00
	Executive function (self)	31.48
	Total (self)	79.93
	Apathy (family)	20.93
	Disinhibition (family)	20.60
	Executive function (family)	31.93
	Total (family)	73.50

Pre-DBS Posterior neuropsychological faculties		
**Clinical Score**	**Subscales**	**Values**
**Language**		
Verbal Associative Fluency (FAS) Test	FAS	40.70
	a	17.49
Digit Symbol	digitsym	6.35
**Visual perception**		
Rey–Osterrieth Complex Figure Tests	ROCF copy	26.56
**Memory**		
Rey–Osterrieth Complex Figure Tests (ROCF)	ROCF short-term memory (STM)	11.87
	ROCF long-term memory (LTM)	11.00
Rey Auditory Verbal Learning Tests (RAVLT)	RAVLT1	5.80
	RAVLT5	11.10
	RAVLT learn	44.00
	RAVLT6	4.30
	RAVLT7	9.00
	RAVLT8	8.23

PreDBS Mixed Faculties Scores		
**Clinical Score**	**abbreviation**	Norms
Working Memory	Digit span backward (BWD)	4.30
Working Memory	Arithmetic	10.20
Attention Shifting	Hooper	19.30

Supplementary - Table 3: The distribution of neuropsychological symptoms are differed into dominantly anterior, posterior or mixed faculties. Overall, the cohort's scores distributed evenly and with no significant abnormalities before STN-DBS.

Abbreviations: DBS, deep-brain stimulation; STN, subthalamic nucleus

Supplementary Table 4. Summary of Pre- and Post STN-DBS clinical scores.

A: Pre-STN-DBS Clinical Scores (performed at a mean interval of 2.9±0.77 months before DBS).

Neuropsychological	Speech-Voice	Motor
An overall homogenous distribution of motor symptoms, good LDOPA responsiveness and normal neuropsychological and speech.		
• Patients presented with normal neuropsychology scores pre-DBS.<br>• Psychiatric comorbidities were homogenously nonsignificant pre-DBS.<br>• Patients did not mention any significant neuropsychological differences between ON/OFF states.	• Patients presented with normal speech and voice scores pre-DBS, except 3 scores (VHI E,F,P).<br>• Speech and voice scores given by patients, caregivers, and clinicians did not differ.<br>• UPDRS speech articles significantly improved LEDD.	• Symptom types (tremor, rigidity, akinesia and gait) and severities were homogenously distributed.<br>• ON/OFF/UPDRS IV had the highest correlation with ADL compared to all specific symptom.<br>• LEDD had a significant positive impact.

B: Post-STN-DBS Clinical Scores (done at a mean interval of 16.1±1.4 months after DBS).

Neuropsychological	Speech-Voice	Motor
Significant improvement in all motor scores (except axial signs) and mildly accepted deterioration in neuropsychological (personality, attention & language) and speech (intelligibility) scores. Axial symptoms and voice physical properties seem indifferent to DBS.		
• Mild deterioration in anterior faculties vs undetermined trend in posterior ones.<br>• Personality (FLOPS), Attention (TMT & WCST) and Language (FAS/A) are the most sensitive scores.<br>• NRMS and STN-width (>>β-oscillations) predict milder deterioration.<br>• Post-DBS there is a significant variability of patients/caregivers/clinicians scores.	• Mild deterioration in speech intelligibility with much less significant changed voice physical properties.<br>• 6-Point Scale & VAS 5&6 best assess speech intelligibility and Pitch/GRBAS for voice properties.<br>• Drugs' positive impact is dominant and irreplaceable by DBS.<br>• Post-DBS there is a significant variability of patients/caregivers/clinicians scores.	• The improvement of ON/OFF-UPDRS IV best predicted ADL outcomes, compared to any other symptoms.<br>• All MER properties predict improved motor outcomes except axial signs which are DBS resistant.<br>• β-oscillation best correlated with better ON/OFF improvement.<br>• Drugs maintain an independent positive effect, irreplaceable by DBS.

Supplementary - Table 4A: Summary of Pre-STN-DBS Clinical Scores An overall homogenous distribution of motor symptoms, good LDOPA responsiveness and normal neuropsychological and speech.
Supplementary - Table 4B: Summary of Post-STN-DBS Clinical Scores Significant improvement in all motor scores (except axial signs) and mildly accepted deterioration in Neuropsychological (Personality, Attention and Language) and Speech (Intelligibility) scores. Axial symptoms and voice physical properties seems indifferent to DBS.

Abbreviations: ADL, activity of daily living; DBS, deep-brain stimulation; FAS, phonemic verbal fluency; FLOPS, frontal lobe personality score; GRBAS, grade, roughness, breathiness, asthenia, strain; LEDD, levodopa equivalent daily dose; MER, microelectrode recording; NRMS, normalized root mean square of the neuronal firing rate; STN, subthalamic nucleus; TMT, trail making test; UPDRS, unified Parkinson's disease rating scale; VAS, visual analog scale; VHI, voice handicap index; WCST, Wisconsin card sorting test

Supplementary Table 5. Stereotactic properties (right) and MER properties (Left).

A: Averaged target properties of 198 trajectories

Averaged Target Properties Of 198 Trajectories		
Coordinates/Angels	Left STN	Right STN
X	10.47±0.62	10.20±0.83
Y	-2.66±0.62	-2.53±0.48
Z	-4.55±0.78	-4.57±0.92
Arc	105.76±4.40	72.43±5.40
Ring	66.01±7.14	68.36±3.52
AC-PC Distance	25.8±1.3 (Women-25.3±1.5;Men-26.2±1.1)	

B: MER properties of implanted trajectories

MER Properties of Implanted Trajectories	
NRMS peak	2.44±1.5
STN-width	5.58±1.5
β-oscillatory (DLOR) width	2.58±1.4
DLOR/STN ratio	46.23±10.1%
Occurrence of DLOR	71.30%
ϒ-oscillation decrease	58%
STN-DBS score	5.5±2.8
Active contact within the DLOR	87%

Supplementary - Table 5A: Stereotactic coordinates (X,Y,Z, Arc, Ring) were similar on right and left STN. Both these coordinates and the AC-PC distances (with the normally accepted differences between woman and men) are in accordance with previous studies.
Supplementary - Table 5B: MER properties show that 71.3% of the patients had a β-oscillatory DLOR which typically occupied 46.23±10.1% of the whole STN and with half of the patients (58%) showing a concomitant ϒ-oscillation decrement. The integrative STN-DBS score (composed of NRMS, STN-width and DLOR/STN ratio) was 5.5±2.8. All these MER properties, except the ϒ-oscillation decrease correlated with outcomes (see Figure 2 in the main paper), however differently with regard to motor and non-motor scores. Once the patients achieved a neurological steady state, the neurologists' choice of the "best contact" (blind to any MER or neuroanatomical data) was found to be in 87% of the cases within the dorso-lateral STN, either with or without β-oscillatory activity.

Abbreviations: DBS, deep-brain stimulation; DLOR, dorso-lateral oscillatory region MER, microelectrode recording; NRMS, normalized root mean square of the neuronal firing rate; STN, subthalamic nucleus

Supplementary Table 6. Practical Recommendation for using STN-DBS score.

Step 1: Pre-STN-DBS Scores

Focus on the principal factor during the pre-DBS scoring in a symptom driven approach: Motor/On-Off fluctuation/ADL/Voice/Nψ

- It should take no longer than 1.5-2 hours for which a common ON-duration should suffice.
- Once the 3 modalities were tested at an ON state, have a motor & voice scoring on a true OFF state, in order to evaluate LEDD impact.

**1st factor: Rigidity, Bradykinesia, UPDRS-IV**	**1. Rigidity, Bradykinesia<br>2. UPDRS IV<br>3. Axial**
**2nd factor: Tremor**	**1. UPDRS Tremor scores**
**3rd factor: Speech Intelligibility**	**1. 6 points score<br>2. UPDRS-Speech scores + VHI F**
**4th factor: Voice Physical properties**	**1. GRBAS<br>2. Median pitch**
**5th factor: Anterior Nψ faculties. (Executive functioning, attention & concentration)**	**1. Personality: FLOPS<br>2. Attention: TMT-A**
**6th factor: Posterior Nψ faculties (Memory and Language)**	**1. Verbal memory: RAVLT tests (8!)<br>2. Language: FAS**

Step 2: Expectations.

- Expect favorable outcomes in all motor scores, ON-OFF fluctuations and UPDRS IV but not on axial scores.
- LEDD reduction will be significant but not abolished. It maintain its positive effect mainly upon Speech/Voice, without adverse effects.
- Asses the impact of ON/OFF-UPDRS IV upon ADL. It might become the main surgical objective.
- Axial symptoms most probably remain irresponsive to STN-DBS.

Step 3: Neuroimaging

- Optimize sequences for having STN width > 3 mm (=2 effective contacts!)
- Consider MRI under general anesthesia) for movement reduction.

Step 4: Intra-Operative MER

Aim for a STN-DBS Score of > 2.5 Using >1 electrode/trajectory and With neurological testing

STN-Width > 3mm

β-Oscillatory-DLOR > 1/3 of STN

NRMS > 1.5 at STN penetration

Immediate post-DBS neuroimaging defining the contacts' locations and their relation to the MER.

Step 5: En-route to Steady-State (~ 3-6 months post-DBS)

The 1st contact to activate should be within the β-Oscillatory DLOR !

- Begin with 130–185 Hz, <4 V and 60-200µs
- Before switching contact make sure to optimize the 1st contact and log all changes.

Step 6: 18-24 months post-DBS

- Repeat Step 1 Scores: asses the outcomes & correlation to MER
- Pay attention to adverse effects (Voice/Nψ) and gait disturbances.

Supplementary - Table 6: Practical Recommendation for using STN-DBS score. Pre-STN-DBS steps 1-3: [Step 1]: Begin with a pre STN-DBS score evaluation of the patient in a rather symptom-oriented approach, which emphasizes the 6 main factors: Motor/ON-OFF fluctuation/ADL/Voice/Nψ. Such an evaluation should be done during no more than 1.5-2 hours, beginning on a real ON state and at its end switching to an OFF state in order to evaluate the drug impact pre-DBS. [Step 2]: the clinical scoring can assist in better estimating both the outcomes and the objectives of the intra-operative MER. Patients with dominant axial signs should not be addressed by STN-DBS. [Step 3]: the pre-DBS MRI must be done under general anesthesia in order to optimize the STN identification and, if possible, to define the largest axis with a minimum of 3 mm. Intra-Operative step 4: [Step 4]: the STN-DBS score should be at least > 2.5 and composed of STN-width > 3mm, NRMS > 1.5 and β-oscillations > 1/3. Moreover, for patients with either a dominant tremor, limb bradykinesia or rigidity, the NRMS and STN width seem the best predictors of outcomes, while for patients with dominant ON-OFF fluctuations, it is the β-oscillatory activity. An immediate post-DBS neuroimaging will be done for confirmation of the final location. Post-STN-DBS steps 5-6: [Step 5]: we recommend to chose the contact within the DLOR as the first one to test clinically and to optimize before switching to another one in case of insufficient response. [Step 6]: The same clinical protocol (factor based) should be repeated for evaluation of both the DBS and the remaining LEDD impacts.

Abbreviations: ADL, activity of daily living; DBS, deep brain stimulation; DLOR, dorso-lateral oscillatory region; FAS, phonemic verbal fluency; FLOPS, frontal lobe personality score; GRBAS, grade, roughness, breathiness, asthenia, strain; LEDD, levodopa equivalent daily dose; MER, microelectrode recording; MRI, magnetic resonance imaging; NRMS, normalized root mean square of the neuronal firing rate; RAVLT, Rey auditory verbal learning test; STN, subthalamic nucleus; TMT, trail making test; UPDRS, unified Parkinson's disease rating scale; VAS, visual analog scale; VHI, voice handicap index; WCST, Wisconsin card sorting test

Supplementary Figure 1. Median Pitch is the only score that changes by gender

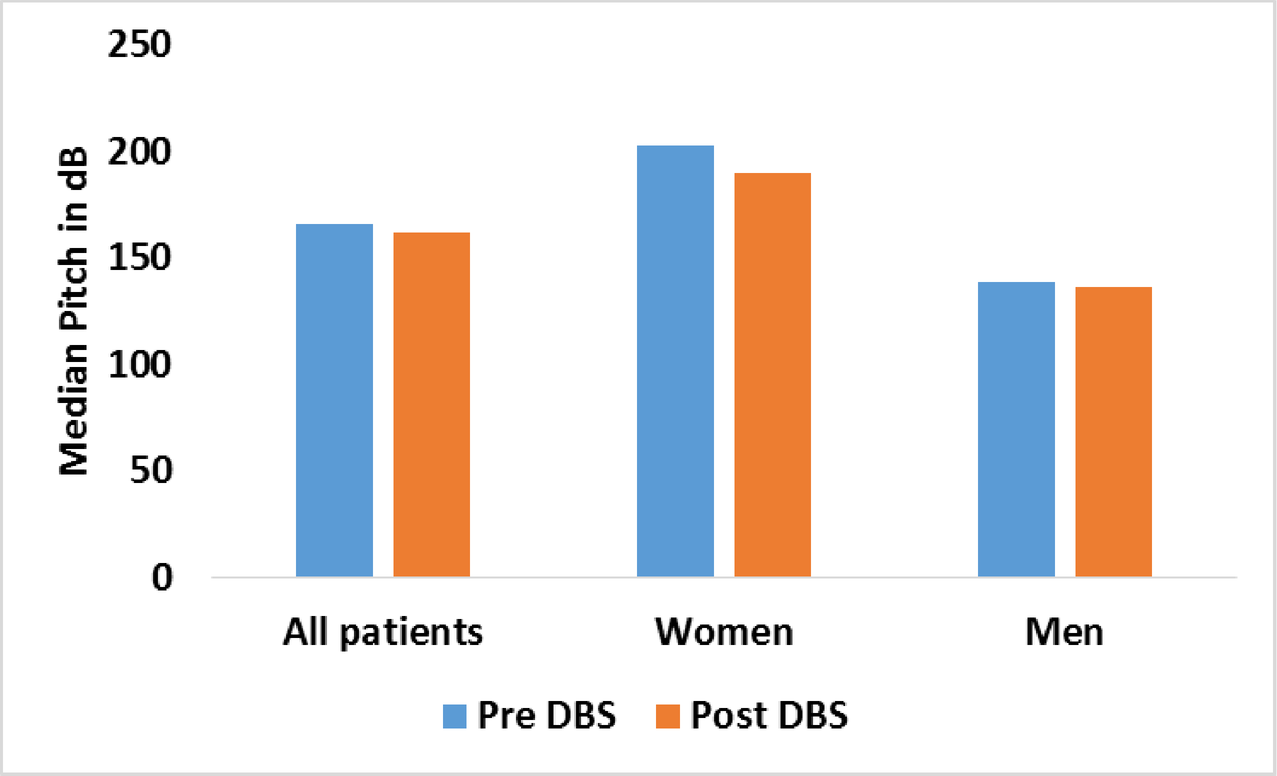

Although the mean median pitch remained within the norms and non-significantly changed (-4.1, NS), we found that it is the only parameter that should be analysed differently by gender. While it decreased in 80% of women (from 203±38 to 189±31Hz [norms: 140-220 dB], n=19) in was almost unchanged in men (from 135.2±23 to 134.5±26 Hz [norms: 85-180 dB], n=29). Comparisons were done using two-way analysis of variance with interaction with gender (male or female). p=0.05.

Abbreviations: DBS, deep brain stimulation
